# Machine Learning Based Method for Impedance Estimation and Unbalance Supply Voltage Detection in Induction Motors

**DOI:** 10.3390/s23187989

**Published:** 2023-09-20

**Authors:** Khaled Laadjal, Acácio M. R. Amaral, Mohamed Sahraoui, Antonio J. Marques Cardoso

**Affiliations:** 1 CISE—Electromechatronic Systems Research Centre, University of Beira Interior, Calçada Fonte do Lameiro, P-6201-001 Covilhã, Portugal; laadjal.khaled1991@gmail.com (K.L.); m.sahraoui@univ-biskra.dz (M.S.); ajmcardoso@ieee.org (A.J.M.C.); 2 Polytechnic Institute of Coimbra, Coimbra Institute of Engineering, Rua Pedro Nunes—Quinta da Nora, P-3030-199 Coimbra, Portugal; 3 GEB Laboratory, Department of Electrical Engineering, Mohamed Khider University, Biskra 07000, Algeria

**Keywords:** three-phase IMs, unbalanced supply voltage (USV), voltage negative factor (VNF), fortescue transform (FT), short time least square Prony’s method (STLSP), impedance estimation, decision tree regressor (DTR) model

## Abstract

Induction motors (IMs) are widely used in industrial applications due to their advantages over other motor types. However, the efficiency and lifespan of IMs can be significantly impacted by operating conditions, especially Unbalanced Supply Voltages (USV), which are common in industrial plants. Detecting and accurately assessing the severity of USV in real-time is crucial to prevent major breakdowns and enhance reliability and safety in industrial facilities. This paper presented a reliable method for precise online detection of USV by monitoring a relevant indicator, denominated by negative voltage factor (NVF), which, in turn, is obtained using the voltage symmetrical components. On the other hand, impedance estimation proves to be fundamental to understand the behavior of motors and identify possible problems. IM impedance affects its performance, namely torque, power factor and efficiency. Furthermore, as the presence of faults or abnormalities is manifested by the modification of the IM impedance, its estimation is particularly useful in this context. This paper proposed two machine learning (ML) models, the first one estimated the IM stator phase impedance, and the second one detected USV conditions. Therefore, the first ML model was capable of estimating the IM phases impedances using just the phase currents with no need for extra sensors, as the currents were used to control the IM. The second ML model required both phase currents and voltages to estimate NVF. The proposed approach used a combination of a Regressor Decision Tree (DTR) model with the Short Time Least Squares Prony (STLSP) technique. The STLSP algorithm was used to create the datasets that will be used in the training and testing phase of the DTR model, being crucial in the creation of both features and targets. After the training phase, the STLSP technique was again used on completely new data to obtain the DTR model inputs, from which the ML models can estimate desired physical quantities (phases impedance or NVF).

## 1. Introduction

In today’s industrial landscape, three-phase induction motors (IMs) dominate, accounting for over 85% of all electric motors utilization [1,2,3,4]. Their widespread adoption stems from their reliability, ease of design, high performance, and ability to handle heavy loads, making them suitable for various applications across manufacturing, processing, power systems, transportation, and more. Despite their benefits, IMs operate in challenging mechanical and electrical environments, rendering them susceptible to multiple stator and/or rotor faults.

One particularly common electrical issue encountered in industrial plants is unbalanced supply voltages (USV), which can disproportionately impact IMs compared to other electrical equipment. Even minor USV can result in significant unbalanced currents due to the relatively low negative sequence impedance, leading to various detrimental effects. These effects include increased heating, elevated losses, vibrations, acoustic noises, reduced torque output, and, ultimately, a shortened lifespan for IMs. Recognizing the potential damages caused by USV, different standards have been established to define permissible limits for this phenomenon. Notable standards include those set by NEMA [5], IEEE, and IEC, each with its own set of considerations [6,7,8]. These standards aim to mitigate the adverse impacts of USV on IMs and ensure their optimal operation.

Unbalanced supply voltages (USV) in industrial power systems can arise from various factors, with some of the most common causes being highlighted in [9]. These include malfunctioning power factor correction equipment, unevenly distributed single-phase loads within the same power system, and open-circuits in the primary distribution system. The investigation of USV has been extensively explored in research papers, focusing on identifying its root causes and examining its impact on electrical machines to establish acceptable tolerance levels.

The dynamics of induction motors are highly intricate, emphasizing the need for a controller capable of robust control considering these dynamics. Induction motor controllers play a vital role in ensuring the protection and supervision of electromechanical systems [5,10]. To fulfill these functions effectively, it becomes imperative to comprehend the dynamic physical model of induction motors. Accurate dynamics are obtained by applying the fundamental principles of physics. These dynamic models rely on physical parameters such as currents, voltages, speed, fluxes, inductances, and resistances, which are directly or indirectly monitored through sensors or estimators. However, due to operational conditions and the presence of noise, achieving precise measurements of some of these values can be challenging. Estimating the impedance of induction motors is a crucial aspect of motor analysis and control in the field of electrical engineering. Accurate knowledge of the motor’s impedance helps in various applications, such as motor protection, fault diagnosis, and control system design [11].

Several techniques and approaches have been developed for impedance estimation of induction motors. One commonly used method is the Extended Kalman Filter (EKF) approach, which combines the motor mathematical model with measured data to estimate the motor parameters, including impedance [5,11]. This approach is discussed through a comprehensive formulation of the EKF algorithm for impedance estimation, and its effectiveness is validated through experimental results.

Signal processing techniques play a crucial role in reducing noise and extracting meaningful features from raw data. In this field, various time domain feature methods [12,13,14], such as Kernel Density Estimation (KDE), Root Mean Square (RMS), Crest Factor, Crest-Crest Value, and Kurtosis, are commonly employed to quantify characteristics. Additionally, frequency domain features [15], obtained through Fourier transformation, and time-frequency features derived from Wavelet Packet Transform (WPT) [16], are widely utilized as indicators for subsequent analysis. Other signal processing methods, including Empirical Mode Decomposition (EMD) [17,18], Intrinsic Mode Function (IMF), Discrete Wavelet Transform (DWT), Hilbert Huang Transform (HHT) [18,19], Wavelet Transform (WT) [20], and Principal Component Analysis (PCA) [21], are also employed for effective signal processing.

After employing signal processing and feature extraction methods, various classification techniques are utilized to identify flaws based on the extracted characteristics. Support Vector Machine (SVM), Artificial Neural Networks (ANN), Wavelet Neural Networks (WNN) [19], dynamic neural networks, and fuzzy inference are commonly employed in this context. Researchers have employed different approaches to leverage these classification techniques. For instance, Ref. [18] utilized Hilbert Huang Transform (HHT) to extract features from marginal spectrum vibration signals, followed by SVM classification using Window Marginal Spectrum Clustering (WMSC) for defect identification. In [22], the statistical locally linear embedding approach was employed to obtain low-dimensional characteristics from high-dimensional data extracted through time domain, frequency domain, and Empirical Mode Decomposition (EMD) techniques. The classifiers utilized in that study were regression trees, the K-nearest-neighbor classifier, and SVM.

In their research, the authors in [23] utilized the Gaussian–Bernoulli Deep Boltzmann Machine (GDBM) to analyze and learn from statistical characteristics extracted from the time domain, frequency domain, and time-frequency domain. The GDBM was also selected as the classifier in their study. On the other hand, in [24], the reported work is focused on optimizing the classifier’s performance by employing a multi-stage feature selection technique to identify the most relevant set of characteristics. Both studies emphasize the importance of feature extraction and selection phases in their respective approaches.

While defect identification techniques offer valuable insights, they do possess certain limitations. Firstly, effectively applying noise reduction and feature extraction methods to real-world challenges requires specialized knowledge in signal processing. Each unique condition may necessitate the use of specific signal processing techniques that rely on expertise in signal analysis and mathematics.

Secondly, the performance of classifiers heavily relies on the quality and relevance of the features extracted from time series signals. While accurate and informative features contribute to accurate identification and decision-making, the presence of confusing or irrelevant features can lead the model astray.

Thirdly, it is important to acknowledge that feature extraction approaches inevitably result in some loss of information. This loss may include the temporal coherence of time series data, which is a significant aspect that should not be disregarded when interpreting and analyzing the results.

This paper proposed two simple solutions with reduced computational cost, which use ML algorithms to estimate the IM phases impedances and detect the USV condition. It should be noted that the impedance estimation does not require the introduction of extra sensors. On the other hand, the detection of the USV condition does not require the computation of the voltage-symmetrical components, which makes the solution simpler and computationally lighter.

## 2. The Proposed USV Fault Detection

The objective was to develop a dependable and measurable indicator that enables quick and real-time detection of Unbalanced Supply Voltage (USV), facilitating prompt actions to safeguard three-phase induction motors. The concept proposed takes inspiration from the examination of voltage imbalances in power network analysis. Specifically, the Voltage Unbalance Factor (VUF) is defined as the ratio between the negative and positive symmetrical components of voltages [25,26]. To emphasize its derivation from the negative sequence, it will be referred as the Negative Voltage Unbalance Factor (NVUF):

(1) $$NVF=NVUF=\left|\frac{V_{N}}{V_{P}}\right|$$

The symmetrical components of voltage are determined through the widely used Fortescue Transform (FT). By applying the FT to the three-phase unbalanced supply voltages (Va, Vb, Vc) of an induction motor, three symmetrical components are obtained: positive (V_P_ or direct), negative (V_N_ or inverse), and zero (V_Z_ or homopolar). These symmetrical components can be expressed in matrix form as follows:

(2) $$\left[\begin{array}{c} V_{P}\\ V_{N}\\ V_{Z} \end{array}\right]=\frac{1}{3}\times \left[\begin{array}{ccc} 1 & a & a^{2}\\ 1 & a^{2} & a\\ 1 & 1 & 1 \end{array}\right]\times \left[\begin{array}{c} V_{a}\\ V_{b}\\ V_{c} \end{array}\right]$$

where $a=e^{\left(2\times \pi \times j/3\right)}$

In the case of balanced supply voltages, only the positive symmetrical component is present, while the negative and zero components remain zero. However, in the event of Unbalanced Supply Voltage (USV), the negative symmetrical components emerge. Therefore, the degree of USV can be assessed by utilizing the NVUF factor defined in Equation (1) [27].

As mentioned earlier, the presence of Unbalanced Supply Voltage (USV) in induction motors results in an imbalance in the line currents, which, in turn, leads to an imbalance in the stator winding impedances. Therefore, the proposed approach involves calculating the symmetrical components associated with both the line stator currents and the stator winding impedances. This allows for the determination of the phase impedances, Negative Current Factor (NCF), and Negative Impedance Factor (NIF), according to the following definitions [28,29]:

(3) $$Z_{ABC}=\frac{V_{ABC}(1fs)}{I_{ABC}(1fs)}$$

(4) $$NCF=\left|\frac{I_{N}}{I_{P}}\right|$$

(5) $$NIF=\left|\frac{Z_{N}}{Z_{P}}\right|$$

**Figure 1:**
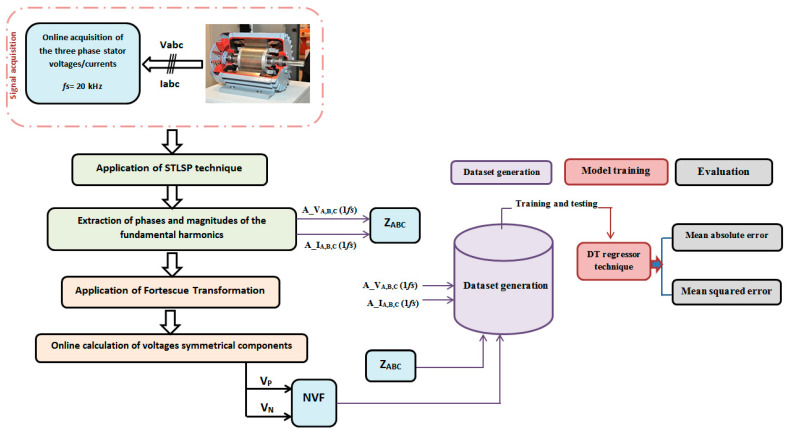
General scheme of the proposed strategy.

The central aspect of the proposed concept is the precise estimation and monitoring of the fundamental harmonics associated with voltages and currents. These harmonics are utilized to compute the necessary symmetrical components, which, in turn, are employed to determine various factors. Consequently, the proposed method can be outlined by the following sequential steps, illustrated in Figure 1 for better clarity and organization:

***Step 01***: Acquisition of the three-phase currents and voltages $\left(V_{a},V_{b},V_{c},I_{a},I_{b},I_{c}\right)$;

***Step 02***: Extraction of fundamental harmonics (magnitudes and phase angles) associated with three-phase voltages and currents $\left(V_{a.1fs},V_{b.1fs},V_{c.1fs},I_{a.1fs},I_{b.1fs},I_{c.1fs}\right)$. This can be achieved using the STLSP method. This method is a high-resolution signal processing technique that accurately estimates and tracks all attributes (frequency, amplitude, phase, and damping factor) of any harmonics from a short data record signal. This capability allows for the consideration of the non-stationary nature of the problem [30]. To enhance the results and mitigate the influence of certain features, a preprocessing step is necessary for the acquired signals. This involves adjusting data acquisition parameters, applying filters, removing DC components, and down-sampling [30,31]. The linear prediction parameters, represented as *a_k_*, are determined to best fit the observed data. Subsequently, these linear prediction parameters are utilized to create a characteristic polynomial with roots, represented as m_k_, using the following approach:

(6) $$f(m)=\sum_{k=0}^{P}\left(a_{k}\times m^{\left(P-k\right)}\right)$$

Consequently, the damping factor and frequency can be obtained directly from the roots, *m_k_*, of Equation (6):

(7) αk=ln⁡mkTsandfk=12×π×Ts⁡tanIm⁡mkRe⁡mk−1

Finally, the roots *m_k_* are utilized to write the *P* equations of (6) in a matrix form as:

(8) $$\left[\begin{array}{cccc} 1 & 1 & \cdots & 1\\ m_{1} & m_{2} & \cdots & m_{P}\\ \vdots & \vdots & \vdots & \vdots \\ m_{1}^{P-1} & m_{2}^{P-1} & \cdots & m_{P}^{P-1} \end{array}\right]\times \left[\begin{array}{c} w_{1}\\ \vdots \\ w_{P} \end{array}\right]=\left[\begin{array}{c} y\left(1\right)\\ \vdots \\ y\left(P\right) \end{array}\right]$$

The complex parameters m_k_ can be obtained by solving Equation (8), which allows for the determination of the exponential amplitudes *A_k_* and phase angles $\phi_{k}$ using the following relationships:

(9) Ak=wkandϕk=⁡tanIm⁡wkRe⁡wk−1

On the practical side, the number of available data samples typically exceeds the number of unknown parameters (N > 2P). In the case of an over-determined dataset, the linear difference can be expressed as follows [30]:

(10) $$\sum_{k=0}^{P}\left(a_{k}\times y\left[n-k\right]\right)=\varepsilon \left[n\right]$$

The available *N* data samples are used to rewrite (10) in a matrix form:

(11) $$\left[\begin{array}{ccc} y\left[P\right] & \ldots & y\left[1\right]\\ \vdots & \ddots & \vdots \\ y\left[N-1\right] & \ldots & y\left[N-P\right] \end{array}\right]\times \left[\begin{array}{c} a_{1}\\ \vdots \\ a_{P} \end{array}\right]=-\left[\begin{array}{c} y\left[P+1\right]\\ \vdots \\ y\left(N\right) \end{array}\right]$$

The unknown parameter vector *a_k_* is chosen to minimize the total squared error of linear prediction. This minimization task can be effectively solved using the least square method. Similarly, the estimation of the complex parameters *w_k_* can be transformed into a linear least square procedure.

(12) M×W⁡=C

with:

$$M=\left[\begin{array}{ccc} 1 & \cdots & 1\\ m_{1} & \cdots & m_{P}\\ \vdots & \cdots & \vdots \\ m_{1}^{N-1} & \cdots & m_{P}^{N-1} \end{array}\right],\;W=\left[\begin{array}{c} w_{1}\\ \vdots \\ w_{P} \end{array}\right],\;C=\left[\begin{array}{c} y\left(1\right)\\ \vdots \\ y\left(N\right) \end{array}\right]$$

***Step 03***: Calculation of the symmetrical components related to the supply voltages and stator currents ($V_{1fs}^{P},V_{1fs}^{N},V_{1fs}^{Z},I_{1fs}^{P},I_{1fs}^{N},I_{1fs}^{Z}$);

***Step 04***: Calculation of the symmetrical components related to the stator winding voltage;

***Step 05***: Calculation of the Negative Voltage Unbalance Factor (NVUF).

## 3. Experimental Configuration

**Figure 2:**
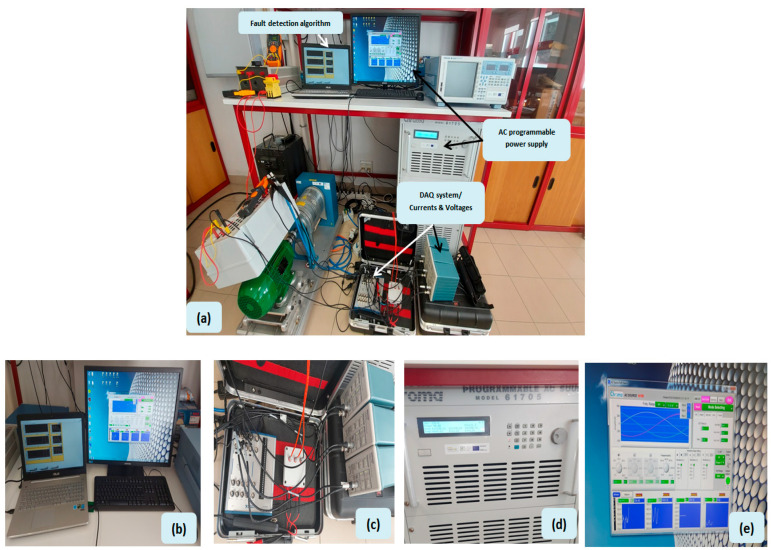
(**a**) Experimental test bench; (**b**) the fault detection algorithm; (**c**) the acquisition system; (**d**) AC programmable power supply; (**e**) AC power supply platform.

In order to generate the necessary data set for the training and validation stages of the ML models, it was necessary to build the experimental configuration represented in Figure 2. This configuration guarantees the reproduction of different operating conditions.

The experimental setup employed for this purpose primarily comprised a three-phase 400 V-50 Hz power supply and a Y-connected, four-pole squirrel-cage induction motor (refer to Table 1 for motor specifications). To facilitate measurements, current transducers utilizing hall-effect technology were utilized, along with a data-acquisition system. Additionally, a remote station was employed to generate voltage unbalance (see Figure 2). By subjecting the unbalance factors to different USV levels and diverse operating conditions, this analysis aimed to provide insights into the performance of ML algorithms.

To create dataset for training and testing the ML models, an algorithm was initially developed to generate the NVUF using Matlab code. Subsequently, the algorithm was integrated into the Lab-VIEW software using the Matlab script mode. The remaining steps of the proposed method, including filtering, down-sampling, and offset removal, were directly performed using Lab-VIEW palettes. For data acquisition, IM voltage and current signals were captured using a NI USB-6366 Series data acquisition card, operating at a sampling frequency of 20 kHz. These steps are executed continuously, enabling real-time monitoring of the target indicators and various motor parameters, such as voltages, currents, impedances, and symmetrical components.

## 4. Machine Learning Algorithm for Estimating Phase Degradation Level

As mentioned in the previous sections, the Voltage Unbalance Condition (VUC) affects the performance of the Induction Motor (IM), causing heating, oscillating torque, and mechanical stresses, which, in turn, can lead to a short circuit between turns and, thus, reduce the IM useful life. Therefore, it is of paramount importance the estimation of the phase impedance (Z_ph_) to assess its level of degradation.

This section presents a solution that is able to estimate the Z_ph_ values without adding extra sensors, so that it is possible to optimize the fault detection scheme. Hence, the main functional requirement for the design of this solution is to use physical quantities that make Z_ph_ estimation possible while simultaneously not requiring the use of extra sensors. The physical quantities that meet the above requirements are the phase currents (I_ph_) since they are required for the voltage source inverter control (VSIC).

In order to estimate Z_ph_ using just I_ph_, without resorting to phase voltages (V_ph_), it is necessary to use machine learning (ML) algorithms. ML algorithms can be subdivided into three types: supervised learning (SML), unsupervised learning (UML), and reinforcement learning (RML). The ML algorithms that will be used to estimate Z_ph_ fall into the first category (SML), as the training data covers not only the inputs but also the outputs. SML algorithms learn to identify patterns between inputs (features) and outputs (target), which gives them the ability to make predictions on new data. Therefore, a model capable of predicting the system’s response is generated. Equation (13) represents a generic SML model.

(13) $$y=f\left(X_{i},\;K_{j}\right)+E$$

where:

- y represents the dependent variable, target or output. In the problem under analysis, Z_ph_ represent the dependent variable;
- Xi represent the i independent variable, feature or input. In the problem under analysis, I_ph_ represent the independent variable;
- Kj represent the model’s parameters. The model’s parameters are estimated during the training phase;
- j stands for the number of parameters, and represents one of the model’s hyper parameters that can be configured to improve the final response. The model’s hyper parameters can be adjusted through a process denominated by ML model tuning;
- E symbolizes the error between the model predictions and the actual response.

SML models can be subdivided into parametric and non-parametric ones. The parametric SML models (PSMLM) use a predefined function to map the input variables into the output variable. One commonly used PSMLM is the linear regression (LR), which assumes a linear relationship between the features and the target. The non-parametric SML models (NPSMLM) does not make any assumptions about the function that maps the features into the target; therefore, these models do not have a priori a fixed number of parameters before the training phase. One commonly used NPSMLM is decision tree regression (DTR), whose number of parameters varies significantly depending on the size and complexity of the training data set.

It should be noted that the problem under analysis requires the estimation of a continuous value and not the estimation of discrete one as in the solutions proposed in [18,19,22], which is why ML classification models will not be addressed.

In order to design a suitable model for the problem under analysis, the following steps were performed:

1. Dataset creation and feature selection;
2. ML selection;
3. Testing and final evaluation of ML models.

### 4.1. Dataset Creation and Feature Selection

The data set used in training and validation stages of the ML models required the construction of the experimental configuration described in Section 3. This configuration assures the reproduction of different operating conditions, which, in this article, correspond to the different scenarios described in Table 2.

After implementing the previous configuration, the currents and voltages in the three phases of IM were acquired for different scenarios. Afterward, the maximum possible attributes were extracted from both phase voltages (V_ph_) and currents (I_ph_) using the STLSP algorithm, namely:

1. The amplitude of I_ph_ (A_IA, A_IB and A_IC) and V_ph_ (A_VA, A_VB and A_VC) at the converter switching frequency (1*f_s_*);
2. The damping factor (DampF) of I_ph_ and V_ph_ at 1*f_s_*;
3. The phase angle (phasA) of I_ph_ and V_ph_ at 1*f_s_*;
4. The estimated 1*f_s_* of I_ph_ and V_ph_.

#### 4.1.1. Feature Selection

The following step was the identification of the attributes provided by the STLSP algorithm that would be effectively important in the construction of the final dataset. Therefore, regarding the target (Z_ph_), it can be computed as follows:

(14) $$Z_{A}=\frac{A\_\mathrm{VA}}{A\_\mathrm{IA}};\;Z_{B}=\frac{A\_\mathrm{VB}}{A\_\mathrm{IB}};\;Z_{C}=\frac{A\_\mathrm{VC}}{A\_\mathrm{IC}}$$

where ZA, ZB, and ZC represent the Z_ph_ of phases A, B, and C, respectively.

As for the features, and considering the functional requirements presented above, it can be concluded that just attributes associated with I_ph_ should be used. On the other hand, as the performance of ML models depends considerably on the features, it is fundamental to choose the most adequate ones that contribute to a better performance of the ML models. In this regard, it is important to mention that the use of irrelevant features increases the complexity of the ML model and the computation time [32]. Furthermore, it can introduce noise, which can lead to overfitting [33]. In this way, the best features were selected, taking into account those that had a high correlation with the target. For this purpose, Pearson’s correlation coefficient was used. The Pearson correlation (r) between two variables X (feature) and Y (target) can be computed using (15) where n represents the number of samples:

(15) $$r=\frac{n\times \sum \left(X\times Y\right)-\left(\sum X\right)\times \left(\sum Y\right)}{\sqrt{n\times \sum X^{2}-{\left(\sum X\right)}^{2}}\times \sqrt{n\times \sum Y^{2}-{\left(\sum Y\right)}^{2}}}$$

**Figure 3:**
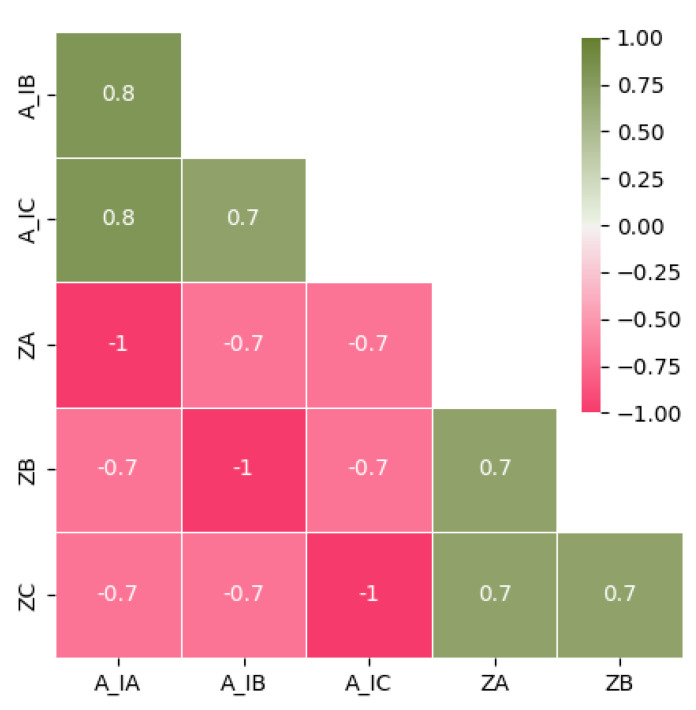
Correlation matrix between the most relevant features (A_IA, A_IB, and A_IC) and the targets (ZA, ZB, and ZC).

After computing r, it was possible to conclude that just A_IA, A_IB, and A_IC present a strong correlation with ZA, ZB, and ZC, as can be seen in Figure 3.

#### 4.1.2. Dataset for ML Model Training and Testing

**Figure 4:**
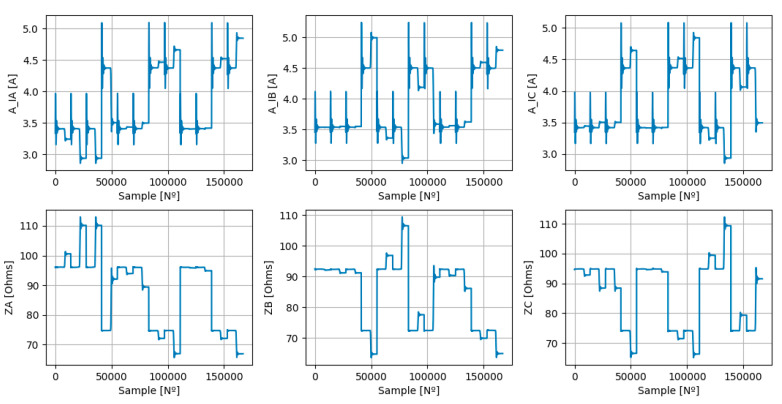
Dataset used for ML models training and testing stages. The features represent the amplitudes of the phase currents at the converter switching frequency (A_IA, A_IB, and A_IC) and the targets represent the phase impedances (ZA, ZB, and ZC).

Finally, it was possible to concatenate all the scenarios described in Table 1 into a single dataset with all relevant features and the targets, as can be seen in Figure 4.

### 4.2. ML Selection

In order to conceive a model that adequately responds to the problem under analysis, two SML models will be evaluated: the LR model and the DTR model.

#### 4.2.1. ML Models

The linear regression (LR) model, as it is a parametric model, imposes a linear function. In this problem, three functions were imposed, one for each target, which are represented in (16).

(16) $$\;\left\{\begin{array}{l} ZA=K_{A1}\times A\_IA+K_{A2}\times A\_IB+K_{A3}\times A\_IC+\beta_{A}\\ ZB=K_{B1}\times A\_IA+K_{B2}\times A\_IB+K_{B3}\times A\_IC+\beta_{B}\\ ZC=K_{C1}\times A\_IA+K_{C2}\times A\_IB+K_{C3}\times A\_IC+\beta_{C} \end{array}\right.$$

where K_ij_ and β_i_ represent the weight of feature j and the bias of target i, respectively.

The LR model estimates both K_ij_ and B_i_ by fitting (16) to the training dataset, and, for this purpose, minimizes the squares of the residuals. The great advantage of LR model is that it is easily interpretable, computationally light, and it is not common to suffer from overfitting. However, the simplicity of the LR model can be a disadvantage, making it less flexible; therefore, its response is more prone to errors, leading to under fitting. For this reason, the performance of a non-parametric model was also evaluated.

**Figure 5:**
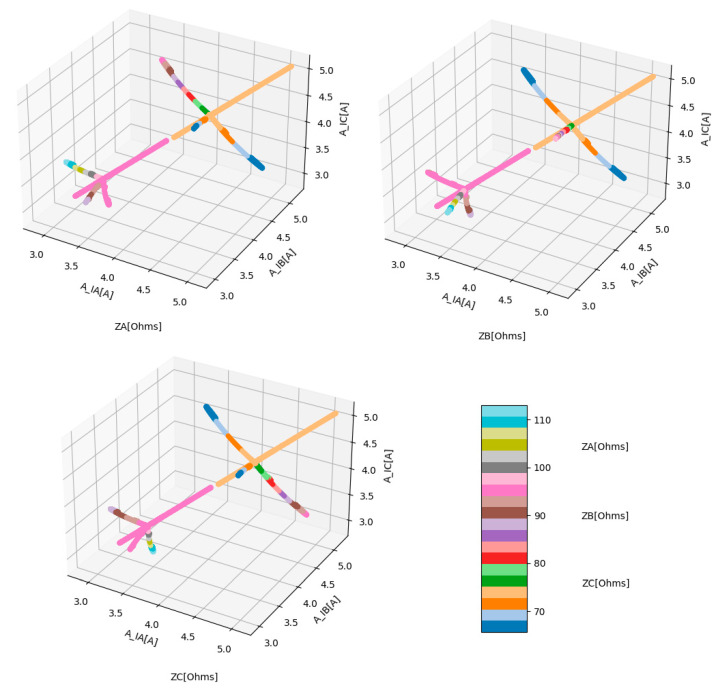
Scatterplots that relate the features (A_IA, A_IB, and A_IC) with the Targets (ZA, ZB, and ZC).

The selected non-parametric model was the DTR, due to its characteristics, namely: it does not require an extremely large number of data, the data are noisy and the output is disjoint. Figure 4 easily corroborates the first two characteristics. In order to show that the dataset output was disjointed, a scatterplot regarding targets is presented below (Figure 5).

The intelligence of the DTR model resides in a set of if-then-else rules that continuously split the data by creating a series of branches, and so, the input data are continuously subdivided into smaller subsets based on the features values until a desired level is reached. The maximum number of levels of DTR model is denominated by the maximum depth tree. 

The DTR model Is composed of a root node, branches, internal nodes, and leaf nodes. The root node, which represents the first node at the top of the tree, has no input branches, but it has output branches that feed subsequent nodes. Internal nodes have input branches and output branches. The first ones come from previous nodes and the second ones feed subsequent nodes. The internal nodes decide how the subdivision of the input data is carried out, and for that, they take into account the threshold value of a specific attribute. The {attribute-“threshold value”} pairs are determined during the training stage. Finally, the leaf nodes, which have no output branches, reproduce the final output, which, in this case, will be the Z_ph_ value.

During the training stage, at each decision node (root and internal nodes), all possible divisions were tested considering all features. For each possible solution, the sum of squares of the residuals was computed. At the end of this process, the division that guarantees the smallest sum of squares of the residues was selected, which defines the best solution, that is the best {attribute-“threshold value”} pair for that specific decision node.

#### 4.2.2. ML Evaluation Metrics

In order to evaluate the performance of both models, two of the most commonly used metrics to evaluate the performance of ML regression models were used: mean absolute error (MAE) and mean squared error (MSE).

(17) $$\mathrm{MAE}=\frac{1}{N}\times \sum_{i=1}^{N}\left|y_{i}-{p_{red}}_{i}\right|$$

(18) $$\mathrm{MSE}=\frac{1}{N}\times \sum_{i=1}^{N}{\left(y_{i}-{p_{red}}_{i}\right)}^{2}$$

where y_i_, p_redi_, and N represent the actual or true value, the predictions, and the total number of samples.

#### 4.2.3. ML Models Comparison

In order to compare the performance of both models, 100 different training and testing datasets were created from a parent dataset. The parent dataset is shown in Figure 4, and each of the training and testing subsets contains random samples of the parent one. For each of the 100 training datasets, an LR and DTR model was generated, which was subsequently evaluated in the corresponding test dataset. Each of the different training datasets contains just 1% of all the data and the remaining 99% is assigned to the corresponding test datasets. 

**Figure 6:**
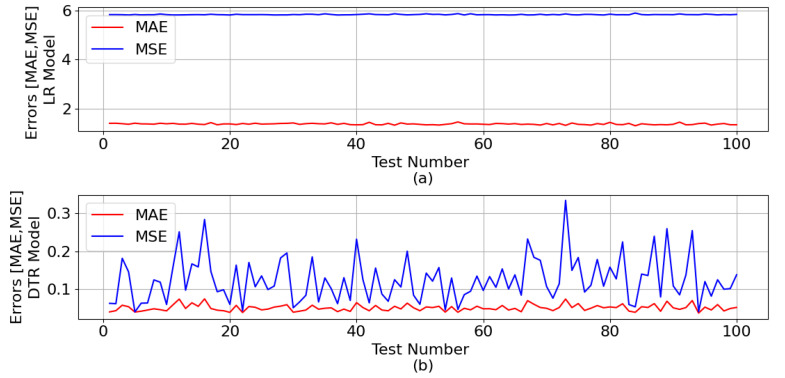
MAE and MSE generated during the ML test phase in relation to the ZA estimation: (**a**) LR model and (**b**) DTR model.

Figure 6 shows the mean absolute error (MAE) and mean squared error (MSE) generated during the test phase, for both ML models, with regard to the ZA estimation.

The mean of all MAE and all MSE for the LR model was 1.381 and 5.827, respectively. As for the DTR model, the means were 0.049 and 0.124, respectively.

**Figure 7:**
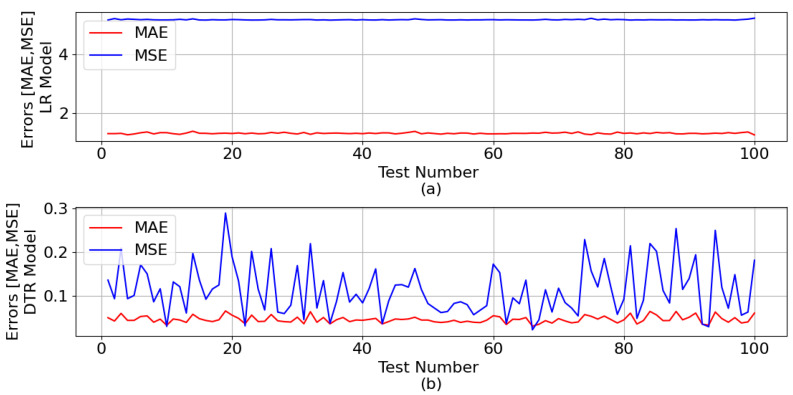
MAE and MSE generated during the ML test phase in relation to the ZB estimation: (**a**) LR model and (**b**) DTR model.

Figure 7 shows the mean absolute error (MAE) and mean squared error (MSE) generated during the test phase, for both ML models, with regard to the ZB estimation.

The mean of all MAE and all MSE for the LR model was 1.307 and 5.181, respectively. As for the DTR model, the means were 0.046 and 0.114, respectively.

**Figure 8:**
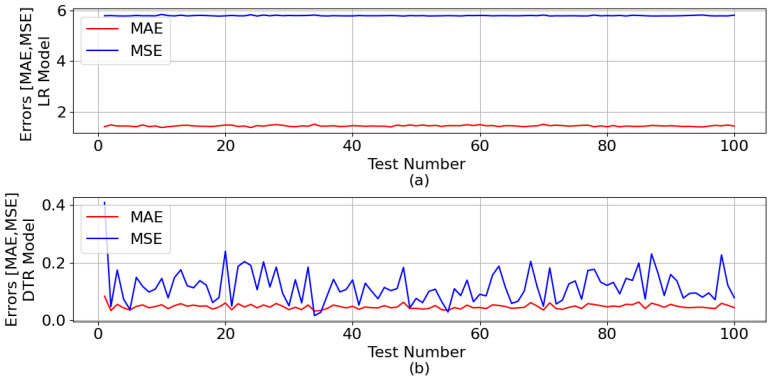
MAE and MSE generated during the ML test phase in relation to the ZC estimation: (**a**) LR model and (**b**) DTR model.

Figure 8 shows the mean absolute error (MAE) and mean squared error (MSE) generated during the test phase, for both ML models, with regard to the ZC estimation.

The mean of all MAE and all MSE for the LR model was 1.435 and 5.786, respectively. As for the DTR model, the means were 0.048 and 0.118, respectively.

As expected, it turned out that for both models, and regarding the estimation of the three impedances, the MSE was greater than the MAE. This can be explained by the fact that the MSE calculates the squared differences between the predicted and actual values; therefore, it tends to amplify the impact of larger errors. The MAE considers only the absolute difference, which results in a more balanced measure.

The greater amplification of larger errors in the MSE also explains why its value in DTR model oscillates so much between the different tests. This phenomenon results from the fact that the DTR model is more sensitive to the training data, as it has a greater tendency to overfitting. In this regard, it is important to mention that the hyper-parameters of the DTR models used in this analysis were not optimized, that is, no limit was imposed on the maximum depth of the tree, which contributes to the described phenomenon.

In any case, the behavior of the DTR model seems to be more appropriate to the problem under analysis since the average errors related to the MAE and MSE are considerably smaller when compared to those of the LR model.

### 4.3. Testing and Final Evaluation of ML Models

In this section, two models were trained and evaluated. The first model used the linear regression model (LRM) described in the previous section and the second one used the decision tree regression model (DTRM) also discussed above. 

**Figure 9:**
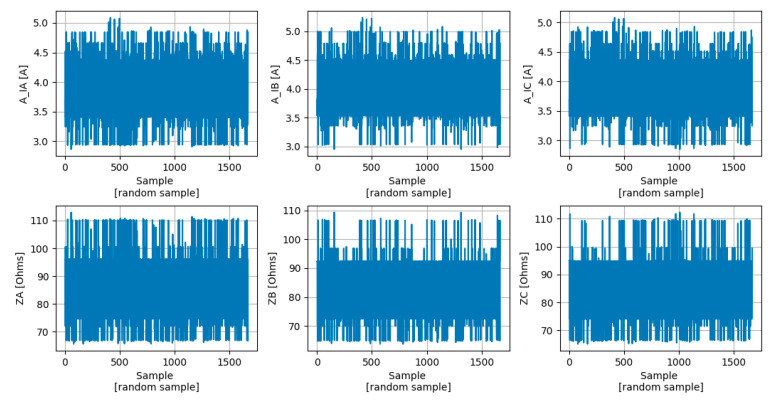
Training dataset (TRDS).

Thus, at first, it is essential to train both models and for that, it is necessary to create a training dataset (TRDS). The TRDS comprises only 1% of the samples in the parent dataset (PADS). The selected samples were randomly chosen, as can be seen in the TRDS that is represented in Figure 9. The test phase of the ML models took into account all data, that is, the PADS (Figure 4).

#### 4.3.1. Linear Regression Model (LRM)

After training the LRM with the TRDS, the functions that relate the targets (ZA, ZB, and ZC) to the features (A_IA, A_IB, and A_IC) were obtained. 

**Figure 10:**
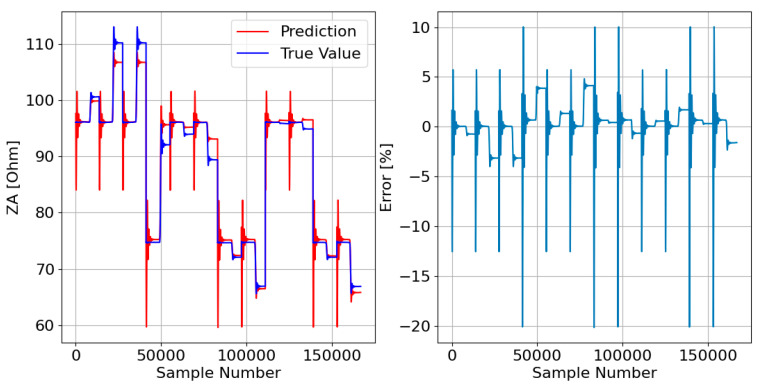
LRM response (function 19) to the PADS: {Features = [A_IA, A_IB, A_IC]; Target = ZA}.

Thus, regarding ZA, function (19) was obtained during training stage, and its response regarding PADS can be observed in Figure 10.

(19) $$\mathrm{ZA}\;≅\;\left(-22.7\right)\times A\_\mathrm{IA}+\left(1.9\right)\times A\_\mathrm{IB}+\left(-0.9\right)\times A\_\mathrm{IC}+169.8$$

The MAE was 1.34 and the MSE was 5.84, which is close to the values obtained in the previous section.

**Figure 11:**
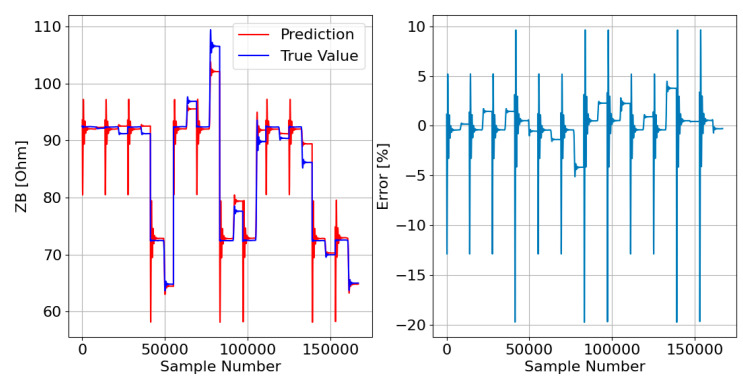
LRM response (function 20) to the PADS: {Features = [A_IA, A_IB, A_IC]; Target = ZB}.

Regarding ZB, function (20) was obtained during training stage, and its response regarding PADS can be seen in Figure 11.

(20) $$\mathrm{ZB}\;≅\;\left(-1.3\right)\times A\_\mathrm{IA}+\left(-20.3\right)\times A\_\mathrm{IB}+\left(1.7\right)\times A\_\mathrm{IC}+162.3$$

The MAE was 1.27 and the MSE was 5.18, which is close to the values obtained in the previous section.

**Figure 12:**
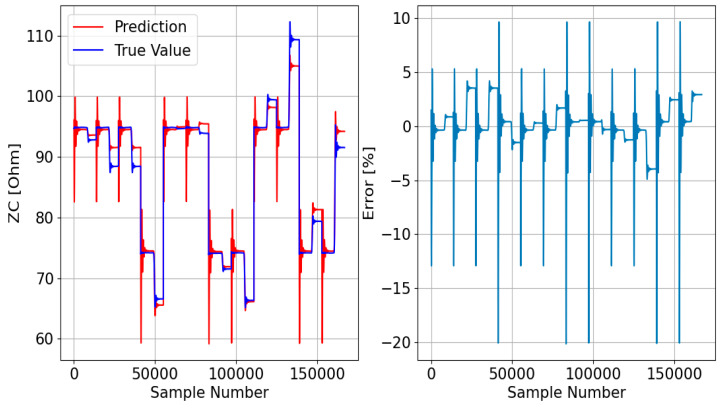
LRM response (function 21) to the PADS: {Features = [A_IA, A_IB, A_IC]; Target = ZC}.

Finally, regarding ZC, function (21) was obtained during training stage, and its response regarding PADS can be seen in Figure 12.

(21) $$\mathrm{ZC}\;≅\;\left(2.3\right)\times A\_\mathrm{IA}+\left(-1.6\right)\times A\_\mathrm{IB}+\left(-21.9\right)\times A\_\mathrm{IC}+166.9$$

The MAE was 1.38 and the MSE was 5.80, which is close to the values obtained in the previous section.

The three LRMs showed a good behavior with an MAE of 1.3 that corresponded to 1.5% of the Z_ph_ mean value and 2% of the lowest Z_ph_. In the subsequent section, the DTRM was trained and evaluated.

#### 4.3.2. Decision Tree Regression Model (DTRM)

As mentioned in Section 4.2.3, the DTRM is sensitive to training data because of its greater tendency to overfitting. Thus, in order to reduce MAE and MSE values, it was initially decided to optimize one of the most important hyper-parameters of the DTRM: the maximum tree depth (MTD). Therefore, after creating the TRDS with only 1% of the samples of PADS, a test dataset (TEDS) was created with the remaining 99% of the samples. The TEDS will be fundamental to apply the pre-pruning technique that optimizes the MTD hyper-parameter.

**Figure 13:**
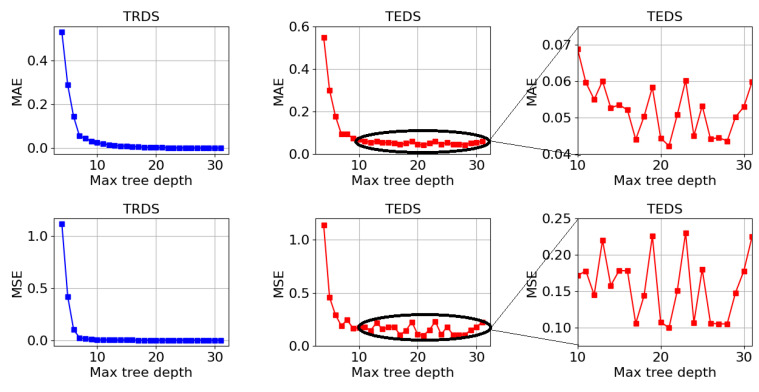
Results of the pre-pruning technique applied to the DTRM of ZA.

The pre-pruning technique consists of identifying the MTD value that produces a DTRM whose response to the TEDS generates the smallest possible errors (MAE and MSE). For this purpose, it is necessary to calculate the error values of different DTRMs, such that each DTRM will have a different MDT value. Thus, at first, it is necessary to train the different DTRMs and simultaneously calculate both MAE and MSE. Subsequently, the response of the trained DTRMs must be evaluated within TEDS and both MAE and MSE must be calculated. Figure 13 shows the application of the pre-pruning technique to the DTRM of ZA.

**Figure 14:**
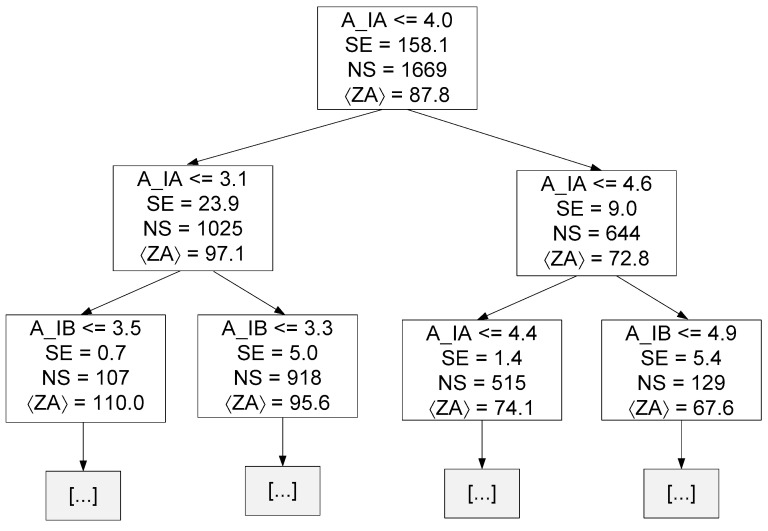
Decision tree resulting from the training phase up to a depth of two (hyper-parameter MDT = 21 and Target = ZA).

The previous figure clearly shows that the best MDT was equal to 21, with the MAE and MSE values being lower than the average value obtained in Section 4.2.3. The previous finding demonstrates that this new pre-pruned DTRM showed an improvement. Figure 14 presents the decision tree {Target = ZA and MDT = 21} resulting from the training stage up to a depth of two.

Where:

- A_IA represents the amplitude of phase A current at 1*f_s_*;
- A_IB represents the amplitude of phase B current at 1*f_s_*;
- SE represents squared error of that specific decision node;
- NS represents the number of samples of that specific decision node;
- 〈ZA〉 represents the mean value of ZA for all samples of that specific decision node.

The pre-pruning technique was also applied to the DTRM of ZB and ZC, and it was found that the best MDT was 21 and 23, respectively. In both cases, the MAE and MSE values were lower than the average value obtained in Section 4.2.3, which demonstrates that both pre-pruned models have improved their behavior.

**Figure 15:**
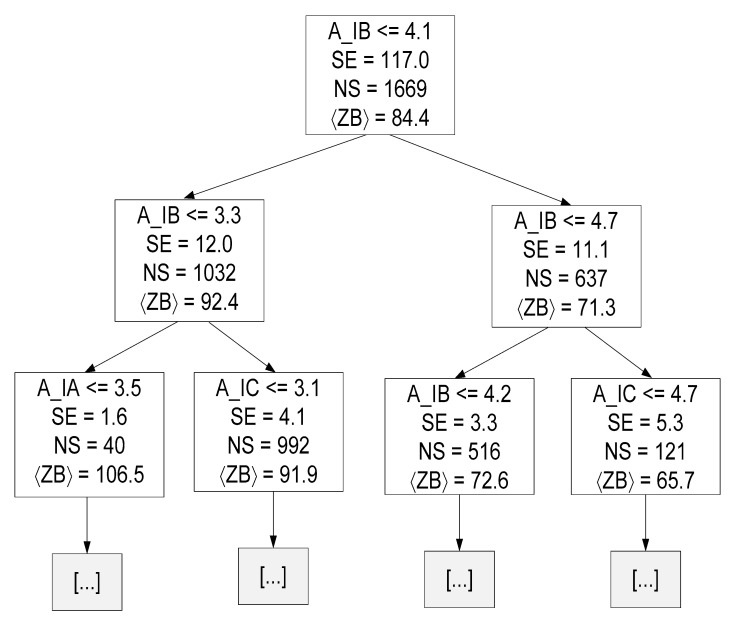
Decision tree resulting from the training phase up to a depth of two (hyper-parameter MDT = 21 and Target = ZB).

Figure 15 presents the decision tree {Target = ZB and MDT = 21} resulting from the training stage up to a depth of two.

Where:

- A_IC represents the amplitude of phase C current at 1*f_s_*;
- 〈ZB〉 represents the mean value of ZB for all samples of that specific decision node.

**Figure 16:**
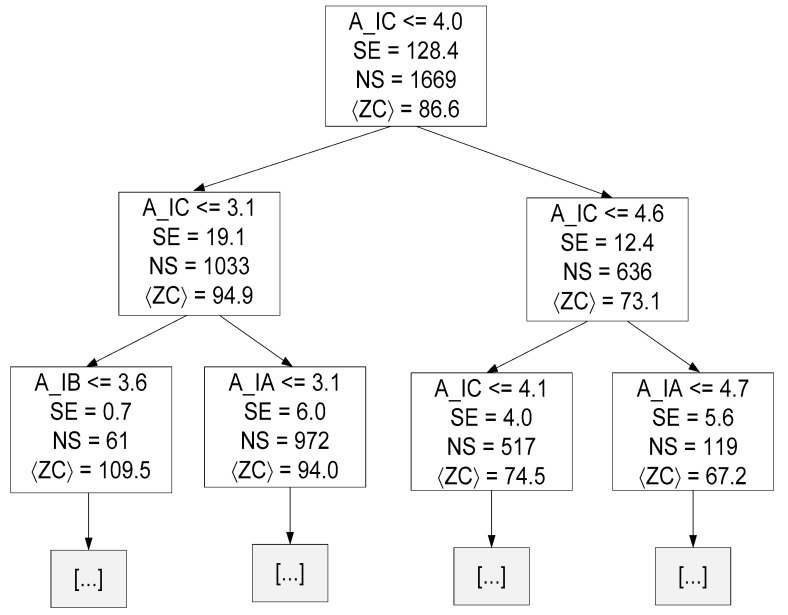
Decision tree resulting from the training phase up to a depth of two (hyper-parameter MDT = 23 and Target = ZC).

Figure 16 presents the decision tree {Target = ZC and MDT = 23} resulting from the training stage up to a depth of two.

Where 〈ZC〉 represents the mean value of ZC for all samples of that specific decision node.

**Figure 17:**
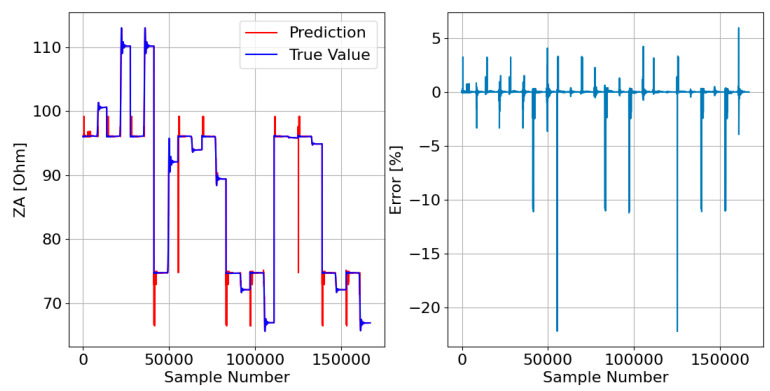
DTRM of ZA (Figure 14) response to the PADS: {Features = [A_IA, A_IB, A_IC], MDT = 21; Target = ZA}.

**Figure 18:**
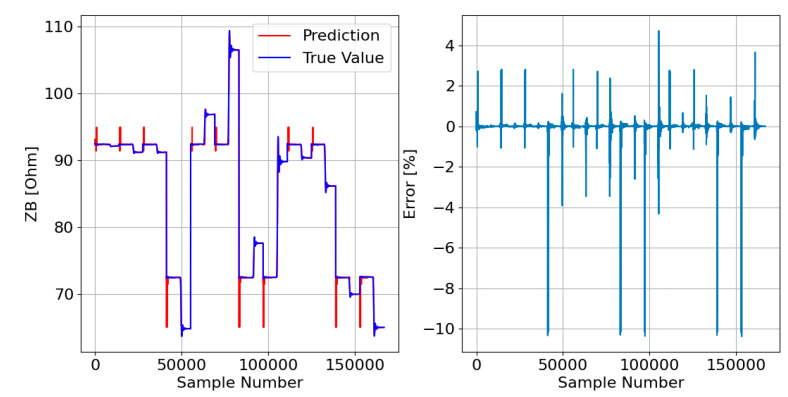
DTRM of ZB (Figure 15) response to the PADS: {Features = [A_IA, A_IB, A_IC], MDT = 21; Target = ZB}.

**Figure 19:**
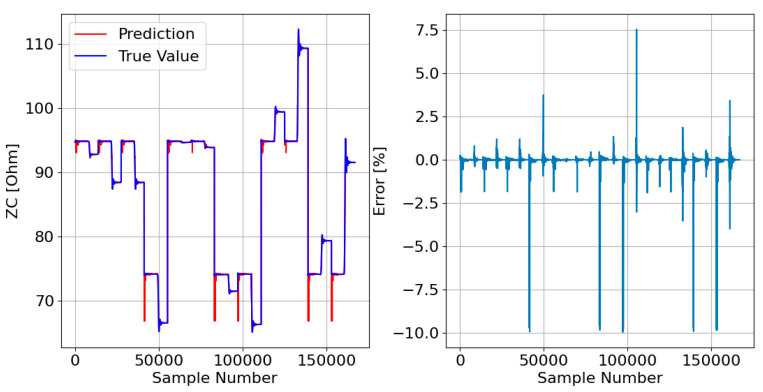
DTRM of ZC (Figure 16) response to the PADS: {Features = [A_IA, A_IB, A_IC], MDT = 23; Target = ZC}.

Afterward, the responses of the three DTRMs to the PADS are presented. Therefore, Figure 17, Figure 18 and Figure 19 show the DTRM of ZA, ZB, and ZC responses, respectively.

#### 4.3.3. Models Comparison

To establish a performance comparison between the two models (DTRM and LRM), Table 3 presents a summary of the errors (MAE and MSE) generated by the models when tested within the PADS.

When comparing the LRM errors of Table 3 with the mean errors (MAE and MSE) presented in Section 4.2.3, it can be seen that they are substantially the same. This observation can be explained by the fact that the LRM model does not have hyper-parameters to adjust and, therefore, cannot improve its performance. With regard to the DTRM, there was an improvement in the model performance for all three phases as MTD hyper-parameter was optimized.

The performance of the LRM was good, and Figure 10, Figure 11 and Figure 12 do not seem to show overfitting which can be explained by the simplicity of the linear models. The LRM output (Z_ph_) represents a simple weighted sum of just three features (A_IA, A_IB, and A_IC). However, despite its simplicity, the model did not seem to show under fitting as it presented similar errors when the training data set was much larger.

Decision tree-based models, on the other hand, tend to overfitting, especially when the data are noisy. However, the results presented in Figure 17, Figure 18 and Figure 19 do not show this problem. The DTRM performed better than the LRM, which can be corroborated by the results in Table 3, which show that the DTRM MAE is thirty times smaller than the LRM MAE, and the DTRM MSE is fifty times smaller than the LRM MSE. It should be noted that the errors presented by the DTRM can be substantially reduced as the training dataset increases in size. However, as the objective is to reduce the probability of overfitting as much as possible, it was decided to train the model on a dataset with only 1% of all data.

Linear and tree-based models are easier to interpret. Through these models, some conclusions can be drawn about the data. For instance, in linear regression, the equation coefficients define the contribution of each feature to the target. Observing Equations (19)–(21), it is possible to conclude that the amplitude of the current corresponding to the phase to be estimated has the greatest contribution. A similar conclusion can be drawn by observing Figure 14, Figure 15 and Figure 16, namely regarding the root node condition.

## 5. Machine Learning Algorithm for Estimating Negative Voltage Factor

As previously mentioned, IMs can be significantly affected by operating conditions, namely by USV, which are quite common in industrial installations. Therefore, it is of paramount importance the development of fault diagnosis techniques that detect and evaluate the USV degree of severity in real time. In this way, more serious failures can be avoided and the reliability and safety of industrial facilities can be increased. Section 2 presents a very reliable indicator that allows the quantification of the degree of severity of the USV, the negative voltage factor (NVF), which requires the calculation of the negative and positive symmetrical components of the stator windings voltage.

The solution presented in this section calculates the value of the NVF without the need to compute the positive and negative symmetric components of the stator winding voltage, it just uses the amplitudes of I_ph_ and V_ph_ at f_sw_. For this purpose, it was necessary to properly train an ML algorithm. For this, the same steps described in Section 4 were performed.

### 5.1. Dataset Creation

**Figure 20:**
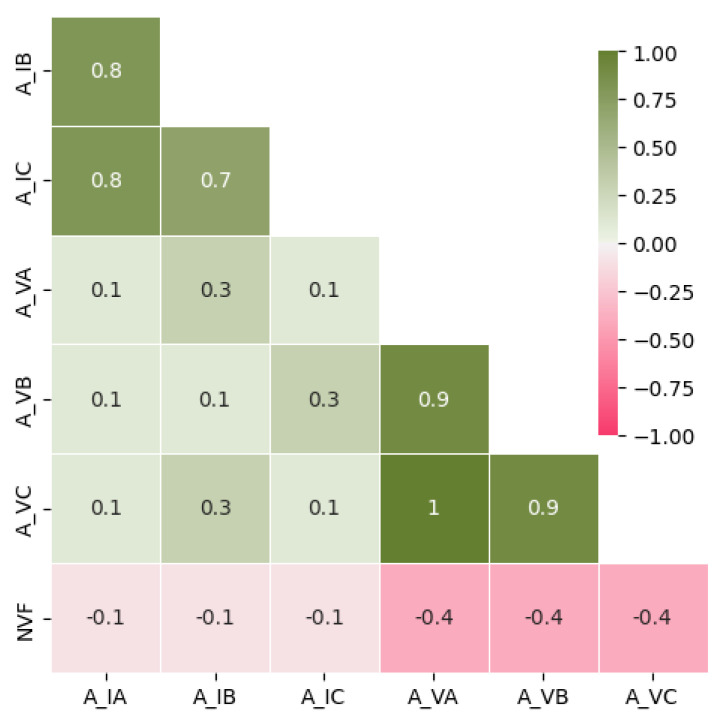
Correlation matrix between the most relevant features (A_VA, A_VB, A_VC, A_IA, A_IB, and A_IC) and the target (NVF).

The dataset used in the training and validation stages of the ML models requires the extraction of a high number of attributes from the I_ph_ and V_ph_, and for this purpose, the experimental configuration described in Section 3 was used. With the help of the STLSP algorithm, it was possible to extract the amplitudes, damping factor, and phase angle of both currents and voltages. To build the dataset, it is necessary to identify the independent variables and the dependent one. The target, which corresponds to the known dependent variable, is the NVF value. Therefore, it is necessary to identify which features are most suitable for the problem under analysis. Hence, Pearson’s correlation coefficient was used, and Figure 20 shows the correlation matrix with the most relevant features.

**Figure 21:**
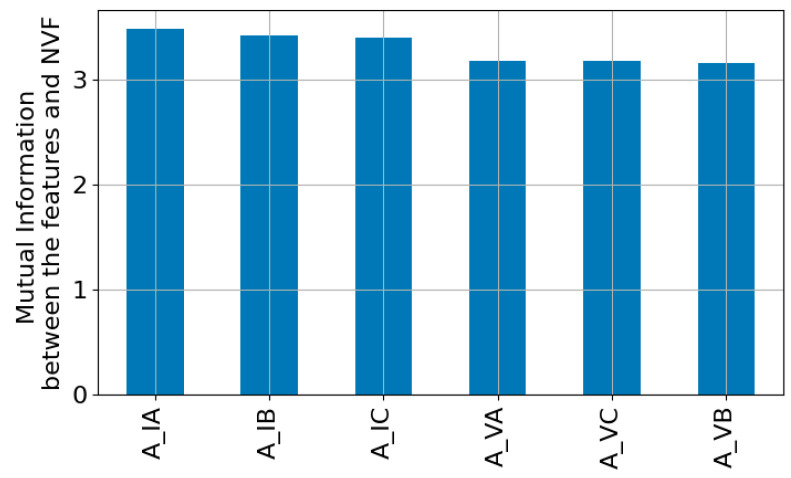
Mutual information between the most relevant features (A_VA, A_VB, A_VC, A_IA, A_IB, and A_IC) and the target (NVF).

The previous figure seems to show that the current amplitudes do not turn out to be relevant features because the value of r is relatively small. However, when calculating mutual information between the I_ph_ amplitudes and NVF, it is possible to perceive their relevance (Figure 21). 

The mutual information between two random variables evaluates the degree of dependence between the two variables, with higher values meaning greater dependence. Therefore, using both feature selection techniques, it is possible to conclude that the most relevant independent variables are the A_VA, A_VB, A_VC, A_IA, A_IB, and A_IC. 

**Figure 22:**
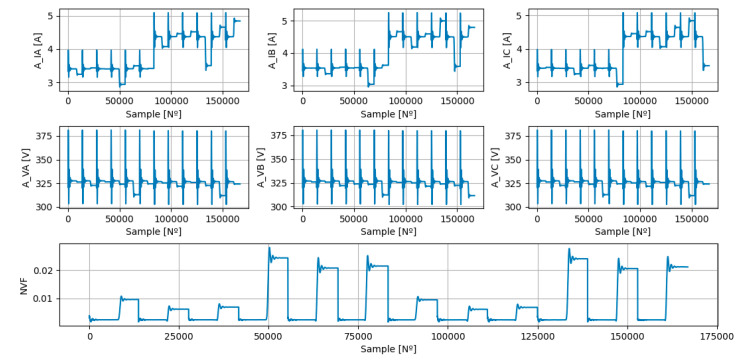
Dataset used for ML models training and testing stages. The features represent the amplitudes of the phase currents and phase voltages at the converter switching frequency (A_IA, A_IB, A_IC, A_VA, A_VB, and A_VC) and the target represent the Negative Voltage Factor (NVF).

Finally, it was possible to build the PADS (Figure 22), which will be used, later, to generate the TRDS and the TEDS.

### 5.2. ML Selection

In this section, two ML models will be evaluated: one parametric, the LRM, and the other non-parametric, the DTRM. 

The evaluation of the model’s performance will be carried out using a very common metric: mean absolute error (MAE). It should be noted that as the NVF value is very small, the mean squared error (MSE) will not be used. In addition, MAE will be calculated in percentage, MAE [%], using the mean NVF value, 〈NVF〉, as a reference:

(22) $$\mathrm{MAE}\left[\%\right]=\frac{\frac{1}{N}\times \sum_{i=1}^{N}\left|y_{i}-{p_{red}}_{i}\right|}{\left\langle \mathrm{NVF}\right\rangle }$$

ML Models Comparison

The LRM is a parametric model; therefore, it imposes a linear function that is represented by Equation (23).

(23) $$\begin{array}{l} \mathrm{NVF}=\mathrm{CA}\_I+\;\mathrm{CA}\_V+\beta \\ \mathrm{CA}\_I=K_{1}\times A\_\mathrm{IA}+K_{2}\times A\_\mathrm{IB}+K_{3}\times A\_\mathrm{IC}\\ \mathrm{CA}\_V=K_{4}\times A\_\mathrm{VA}+K_{5}\times A\_\mathrm{VB}+K_{6}\times A\_\mathrm{VC}\; \end{array}$$

where K_j_ and β represent the weight of feature j and the bias, respectively.

The selected non-parametric model was the DTRM due to the characteristics mentioned in Section 4.2.1.

To compare the performance of both models, 100 different training and testing datasets were created from a parent dataset (Figure 22). For each of the 100 training datasets, an LRM and a DTRM were generated, which were subsequently evaluated on the corresponding test dataset. Each of the different training datasets contained only 1% of all data and the remaining 99% was assigned to the corresponding test datasets.

**Figure 23:**
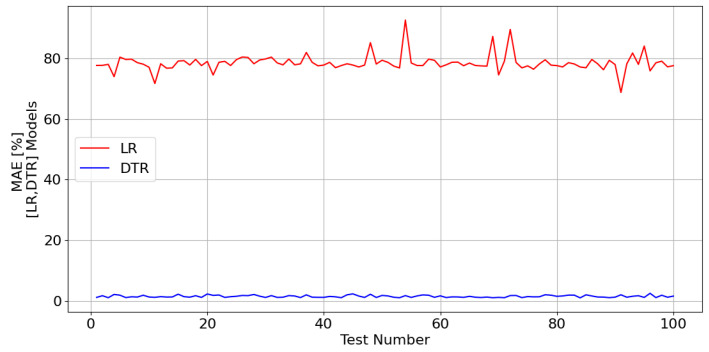
MAE [%] generated during the ML (LR and DTR) models test phase in relation to the NVF estimation.

Figure 23 shows the mean absolute error computed in percentage (MAE [%]) for the LR and DTR models.

The previous figure clearly shows that LRM is not suitable for the problem under analysis. DTRM, on the other hand, revealed a very good performance, which is why it was selected.

### 5.3. Testing and Final Evaluation of the Decision Tree Regression Model

**Figure 24:**
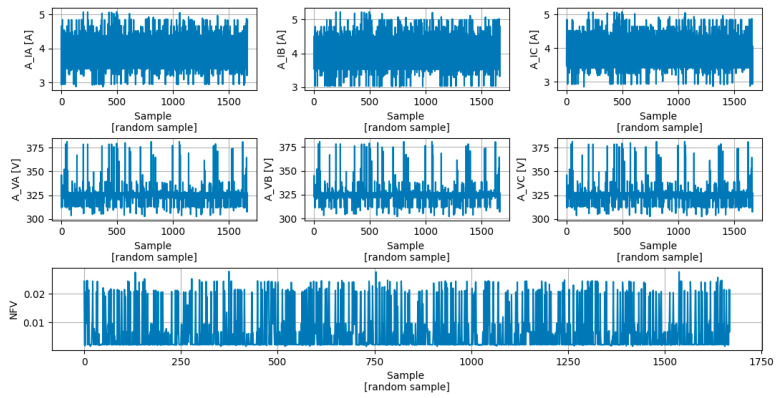
Training dataset (TRDS).

In this section, the DTRM that estimates the NVF value will be presented. However, before presenting the model, it is important to reduce the problems associated with overfitting. For this purpose, two solutions were proposed:

- The TRDS (Figure 24) contains just 1% of the PADS (Figure 22);
- The hyper-parameter MTD was optimized using the pre-pruning technique described in Section 4.3.2. The MDT value that guarantees the smallest MAE [%] in the TEDS is 18.

**Figure 25:**
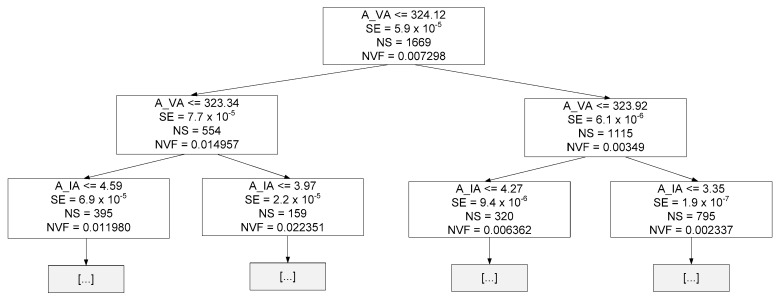
Decision tree resulting from the training phase up to a depth of two (hyper-parameter MDT = 18 and Target = NVF).

After training the DTRM (MDT = 18) using the TRDS, represented in the previous figure, the model in Figure 25 was obtained.

**Figure 26:**
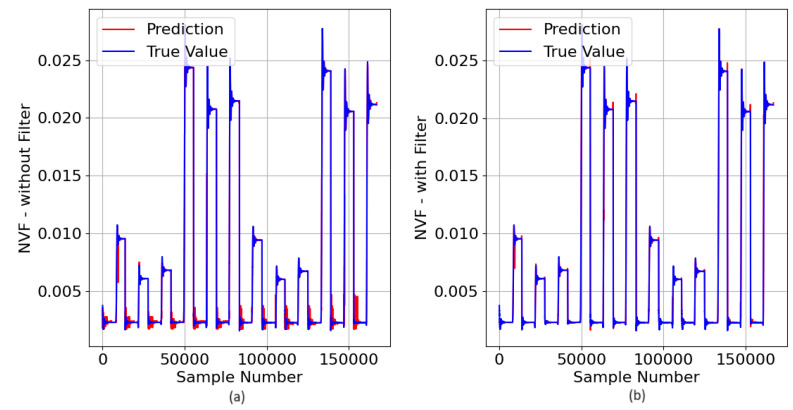
DTRM of NVF (Figure 26) response to the PADS: {Features = [A_IA, A_IB, A_IC, A_VA, A_VB and A_VC], MDT = 18; Target = NVF}: (**a**) without a low pass-filter and (**b**) with low pass-filter.

In the next step, the model (Figure 25) was asked to predict the NVF value in the PADS, with 99% of the PADS data being completely new. The results of the DTRM predictions can be seen in Figure 26, where the true values can also be seen.

Figure 26a shows that the DTRM can predict the NVF value quite well, having presented a MAE [%] of 1.173.

The noise that appears in the DTRM predictions (Figure 26a) occurs in the absence of a fault (NVF = 0.0025) and is, therefore, not critical. However, as it is a high-frequency noise, it can be easily reduced by applying a low-pass filter (Figure 26b).

## 6. Conclusions

The accurate and timely diagnosis of unbalanced supply voltage (USV) conditions plays a crucial role in enabling proactive maintenance and corrective measures, ensuring the reliable operation and safety of industrial applications. It also helps prevent equipment failures, minimize downtime, optimize energy consumption, and enhance overall system performance.

This paper introduced a non-invasive fault diagnosis technique (NIFDT) for induction motors (IMs) that combines the short-time least square Prony’s (STLSP) algorithm with a machine learning (ML) model. The STLSP algorithm processes one of the key attributes of the ML model, which is the amplitudes of the machine’s output voltages and currents at the fundamental frequency. Unsuitable attributes produced by the STLSP model were evaluated using unsupervised feature selection methods and deemed irrelevant. The ML model utilized the remaining attributes, specifically the amplitudes of the voltages and currents. Two ML algorithms were evaluated in this study, and it was demonstrated that the decision tree regressor (DTR) was the most suitable algorithm for the proposed diagnostic technique. The experimental results showed that the DTRM presented a mean absolute error (MAE) of less than 1.2%, which demonstrates the practical applicability of the proposed model. It is noteworthy that the proposed solution did not require the application of the Fortescue Transform, being, therefore, computationally lighter.

The online estimation of the stator impedance holds significance for various objectives, including thermal monitoring, upholding control performance, and facilitating fault detection. Hence, two ML models were proposed for online estimation of the stator impedance using just the phase currents and, therefore, did not require extra sensors. The first approach used the combination of a linear regression model (LRM) with the STLSP technique and the experimental results showed an MAE close to 2%. The second approach used the combination of a DTRM with the STLSP technique, and it showed a better performance with a MAE of less than 0.1%. The proposed approaches demonstrated a notable advantage in terms of reduced sensitivity to parameter deviations when contrasted with alternative methods. This particular advantage becomes more pronounced within a controlled system, especially when confronted with diverse operating conditions such as Inter-Turn Short Circuits (ITSC) and Open Circuit Faults (OCF).

## Figures and Tables

**Table 1:** Induction motor technical parameters.

General	Power [KW]	2.2
	Speed [rpm]	1435
	Frequency [Hz]	50
	Torque [Nm]	14.6
	Voltage [V]	400, Start Connection
	Current [A]	4.6, Start Connection
	Number of poles	4
	Cooling	Closed Motor with external ventilation-IC 411

**Table 2:** Scenarios used in ML models training and testing stages.

Scenarios	Faulty Phase	Load	VUC
1	-	0 Nm	No fault
2		10 Nm	
3–5	A, B and C	0 Nm	5 V
6–8	A, B and C	0 Nm	15 V
9–11	A, B and C	10 Nm	5 V
12–14	A, B and C	10 Nm	15 V

**Table 3:** Performance comparison between DTRM and LRM.

Model	Faulty Phase	MAE	MSE
LRM	A	1.34	5.84
	B	1.27	5.18
	C	1.38	5.80
DTRM	A	0.041	0.098
	B	0.038	0.052
	C	0.041	0.073

## Data Availability

The study did not report any data.
